# Comparative Transcriptomics Reveals a Dual Role of the Epidermal Differentiation Complex in the Skin and the Oesophagus

**DOI:** 10.1111/exd.70181

**Published:** 2025-11-30

**Authors:** Attila Placido Sachslehner, Julia Lachner, Veronika Mlitz, Bahar Golabi, Claudia Hess, Wolfgang Sipos, Leopold Eckhart

**Affiliations:** ^1^ Department of Dermatology Medical University of Vienna Vienna Austria; ^2^ Clinic for Poultry and Fish Medicine, Department for Farm Animals and Veterinary Public Health University of Veterinary Medicine Vienna Vienna Austria; ^3^ Clinical Department for Farm Animals and Herd Management University of Veterinary Medicine Vienna Vienna Austria

## Abstract

The epidermal differentiation complex (EDC) is a cluster of genes implicated in the control of the skin barrier. However, some EDC genes are also expressed at high levels in the human oesophagus. To determine whether the expression of EDC genes in the oesophagus is evolutionarily conserved, we performed comparative transcriptomic analyses of the skin and the oesophagus in humans, mice and chickens. Transcriptomes from public databases and newly generated RNA‐sequencing data of the chicken oesophagus were compared. We found that the EDC of both mammals and birds contains genes that are predominantly expressed in the skin and others that are predominantly expressed in the oesophagus. Cornulin is strongly enriched in the oesophagus of humans and chickens. Similar to small proline‐rich proteins in the human and murine oesophagus, an EDC protein rich in proline is predominantly expressed in the chicken oesophagus. Further oesophagus‐enriched EDC genes are specific to phylogenetic lineages. This study indicates that the EDC plays evolutionarily ancient roles not only in the epidermis of the skin but also in the epithelium of the oesophagus. In line with the dual function of the EDC, dysregulation of EDC gene expression is associated with pathological changes in both stratified epithelia.

## 1 Background

The function of the skin as a barrier against the environment is established by epidermal keratinocytes, which differentiate as they move toward the skin surface. In addition to the accumulation of keratin intermediate filament proteins, alterations in the composition of desmosomes and changes in enzymatic activities [1, 2, 3], a gene cluster known as the epidermal differentiation complex (EDC) is expressed to support the formation of the cornified envelope [4, 5, 6, 7]. Loricrin, small proline‐rich proteins (SPRRs), late cornified envelope (LCE) proteins and others are expressed in homeostatic and stressed epidermis and serve as substrates of transglutaminases (TGMs), which introduce stable intermolecular isopeptide bonds [8]. Further EDC genes such as filaggrin and trichohyalin interact with keratins to modify the cytoskeleton [9, 10]. Cornification (also known as corneoptosis), a form of programmed cell death, completes the differentiation and leaves stable cell ghosts, corneocytes, to build the protective stratum corneum [11, 12].

The epithelium of the oesophagus displays several similarities with the epidermis of the skin, but there are also differences [13, 14]. Like the epidermis, the oesophageal epithelium is a stratified epithelium depending on continuous proliferation of progenitor cells and cell differentiation [15]. However, the outermost layer of the oesophageal epithelium is not cornified and nuclei persist in the superficial cells, a phenomenon known as parakeratosis. The differentiation programme is dysregulated in gastroesophageal reflux disease and upon malignant transformation of epithelial cells resulting in oesophageal adenocarcinoma and squamous cell carcinoma [16, 17, 18, 19]. Mutations of *RHBDF2* cause defects both in the skin and the oesophagus [20]. The normal differentiation of oesophageal epithelial cells is characterised by a gene expression programme which shares genes such as *TGM1* with epidermal differentiation, whereas others such as *MAL* are oesophagus‐specific [21]. Notably, several EDC genes, including *Cornulin* (*CRNN*), *Involucrin* (*IVL*) and *SPRR*s, are expressed at high levels in the oesophagus, where they contribute to epithelial differentiation [22, 23, 24]. These EDC genes are downregulated upon oesophageal inflammation and epithelial transformation [17, 25, 26, 27, 28].

**FIGURE 1:**
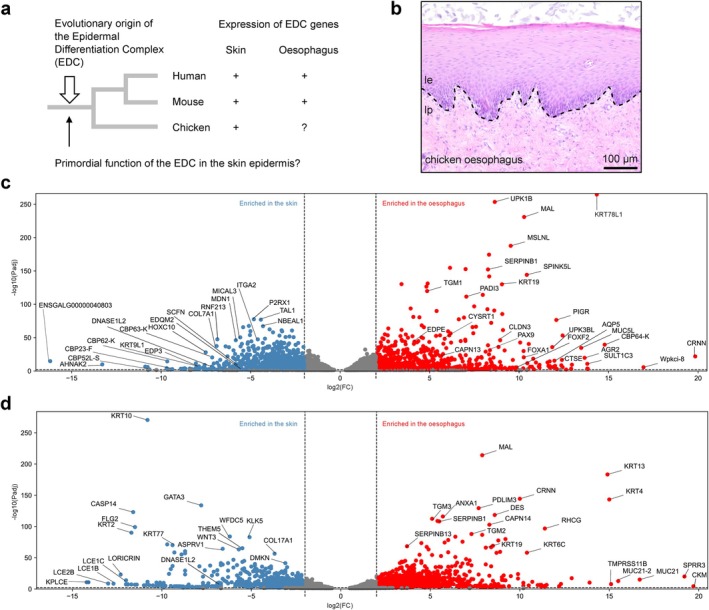
Transcriptome comparison of skin and oesophagus in the chicken. (a) Evolution and expression of the EDC in species investigated in this study. A question mark indicates that the expression pattern of the EDC was unknown in birds prior to this study. (b) Histological analysis by haematoxylin and eosin staining of the chicken oesophagus. le, lamina epithelialis; lp, lamina propria. (c, d) Transcriptome analysis of the skin and the oesophagus in chickens (c) and humans (d). Differences in gene expression levels are shown in volcano plots. Genes with a negative log10 (adjusted *p*‐value, *p*_adj_) > 2 and a log2 (fold change, FC) < −2 (enriched in the skin) or > 2 (enriched in the oesophagus) are coloured in blue and red, respectively.

Previous studies of our and other laboratories have shown that the EDC has evolved in a common ancestor of land‐dwelling vertebrates [29, 30]. Many non‐mammalian EDC genes have been reported to be expressed in the skin and skin appendages [30, 31, 32, 33], whereas only *CRNN* was shown to be expressed in the oesophagus of birds [34, 35]. It is presently unknown whether a larger set of non‐mammalian EDC genes is expressed in the oesophagus, leaving open the question as to whether the EDC has evolved primarily for the regulation of epidermal differentiation, as the name suggests, or for the dual regulation of the epidermis and the oesophageal epithelium and possibly even more epithelia (Figure 1a).

## 2 Questions Addressed

- Is the expression of EDC genes in the oesophagus conserved in a non‐mammalian vertebrate, the chicken?
- Which EDC genes are predominantly expressed in the skin or the oesophagus?

## 3 Experimental Design

### 3.1 Tissue Sampling

Commercial broiler chickens were obtained from VALO Biomedica, Osterholz‐Scharmbeck, Germany. The chickens were sedated by intramuscular injection of a 1:1 mixture of Sedaxylan (20 mg/mL, Dechra Pharmaceuticals) and Narketan (100 mg/mL, Vetoquinol) at the age of 21 days, followed by euthanasia via jugular vein bleeding. Samples were prepared from the uppermost oesophagus region posterior to the pharynx as described previously [36].

### 3.2 Transcriptome Analysis

Freshly dissected tissue samples were stored in RNA‐later (Sigma‐Aldrich) and RNA was extracted with Trizol (VWR) according to the manufacturers' protocols. Three chicken oesophagus samples were subjected to RNA sequencing using published procedures [37]. Details are provided in the Supporting Information. Reads in fastq format were generated using the Illumina bcl2fastq command line tool (Illumina, v2.19.1.403). Raw reads from *n* = 3 samples were uploaded to NCBI GenBank and are available under the Project ID: PRJNA1222808. RNA‐seq data derived from three chicken skin samples were downloaded from NCBI GenBank (Table S1) and converted to fastq‐files with the prefetch and fastq‐dump package from SRA Toolkit, respectively (version: 3.1.1, https://github.com/ncbi/sra‐tools, last accessed on January 7, 2025) and quality‐controlled with FastQC (version 0.12.1, https://www.bioinformatics.babraham.ac.uk/projects/fastqc/, last accessed on January 7, 2025). Using Salmon (version: 1.10.3) [38], the reads were aligned to the Galgal5 reference genome (GenBank accession number GCA_000002315.3), which was previously used for transcriptome analyses of the scutate scales and feathers using the published annotation data for genes of the EDC [39], keratins (KRTs) [40], TGM1 [41] and TGM9 [42]. The quantification of RNA‐seq reads from human and mouse tissues is described in the Supporting Information.

### 3.3 Statistical Analysis

Differential gene expression analysis was performed with PyDESeq2, a module implemented in Python (version: 3.11) [43], which applied the Wald test followed by the Benjamini Hochberg correction for multiple testing to assess statistical significance [44].

## 4 Results

### 4.1 RNA‐Sequencing Analysis Reveals High Expression Levels of Epithelium‐Associated Genes in the Oesophagus of the Chicken

To investigate whether the expression of EDC genes in the oesophagus, as it is known in humans and mice, is conserved in non‐mammalian vertebrates, we chose the chicken as a model (Figure 1a). Histological characterisation by haematoxylin and eosin staining showed that the oesophageal epithelium of the chicken is a stratified squamous epithelium displaying parakeratosis (Figure 1b). This structural organisation and the thickness of more than 200 μm make the chicken oesophageal epithelium similar to its human counterpart [45] and distinguishes it from the mouse oesophageal epithelium, which is orthokeratotic and only about 50 μm thick [46, 47, 48].

The chicken oesophagus was analysed by RNA‐sequencing (RNA‐seq). Several of the genes most abundantly expressed in the chicken oesophagus (Table S2) are markers of stratified epithelia. Among the top ten genes ranked by expression level, the epithelium‐associated genes include one gene of the EDC (*CRNN*), two keratins (*KRT15* and *KRT78L1*) and an ortholog of *SPINK5*, a protease inhibitor implicated in the control of desquamation from the human epidermis and oesophageal epithelium [49, 50].

### 4.2 Comparative Transcriptomics Highlights Skin‐Enriched and Oesophagus‐Enriched Genes in Chickens and Humans

Genes predominantly expressed in either the skin or the oesophagus were identified by comparing the chicken oesophagus transcriptome (see above) to chicken skin transcriptomes available in public databases (Table S3), and by comparing skin and oesophagus transcriptomes of humans (Table S4) and mice (Table S5). The normalised expression levels of all genes expressed in the oesophagus and the skin were subjected to statistical analysis with correction for multiple testing. Using a threshold of differential expression of log2 fold change > 2 and an adjusted *p*‐value (*p*_adj_) < 0.01, the expression of 1262 genes was enriched in the skin of the chicken and the expression of 914 genes was enriched in the oesophagus (Figure 1c, Table S3). Many EDC genes encoding corneous beta‐proteins (CBPs), also known as beta‐keratins, were among the genes with the most pronounced expression bias toward the skin in the chicken (Figure 1c, Figure S1). By contrast, the EDC gene *CRNN* was the most strongly oesophagus‐enriched gene and another EDC gene, *CBP64*, was ranked third according to the ratio of expression in the oesophagus versus the skin (Figure 1c).

The comparative analysis of human skin and oesophagus, based on transcriptome datasets available in public databases, showed that several of the oesophagus‐enriched genes of the chicken are homologues of oesophagus‐enriched genes of humans (Table S4). For example, *CRNN*, *MAL* and *SERPINB1* are predominantly expressed in the oesophagus of both species. On the contrary, *DNASE1L2*, a gene implicated in cornification‐associated DNA degradation [51], was expressed in a skin‐enriched manner in chickens and humans (Figure 1c,d). However, several other genes with tissue‐biassed expression, such as skin‐associated *CASP14* in humans and oesophagus‐associated *MSLNL* of the chicken, lack homologues in the respective other species. Thus, the gene expression profiles of the skin and oesophagus are only partially conserved in the chicken and humans. The murine oesophagus differs substantially from the human and the chicken oesophagus in terms of gene expression (Table S5). Nevertheless, a small subset of genes is oesophagus‐enriched in all three species investigated (Table S6).

### 4.3 Both Humans and Chickens Have EDC Genes That Are Predominantly Expressed in the Skin and Others That Are Predominantly Expressed in the Oesophagus

**FIGURE 2:**
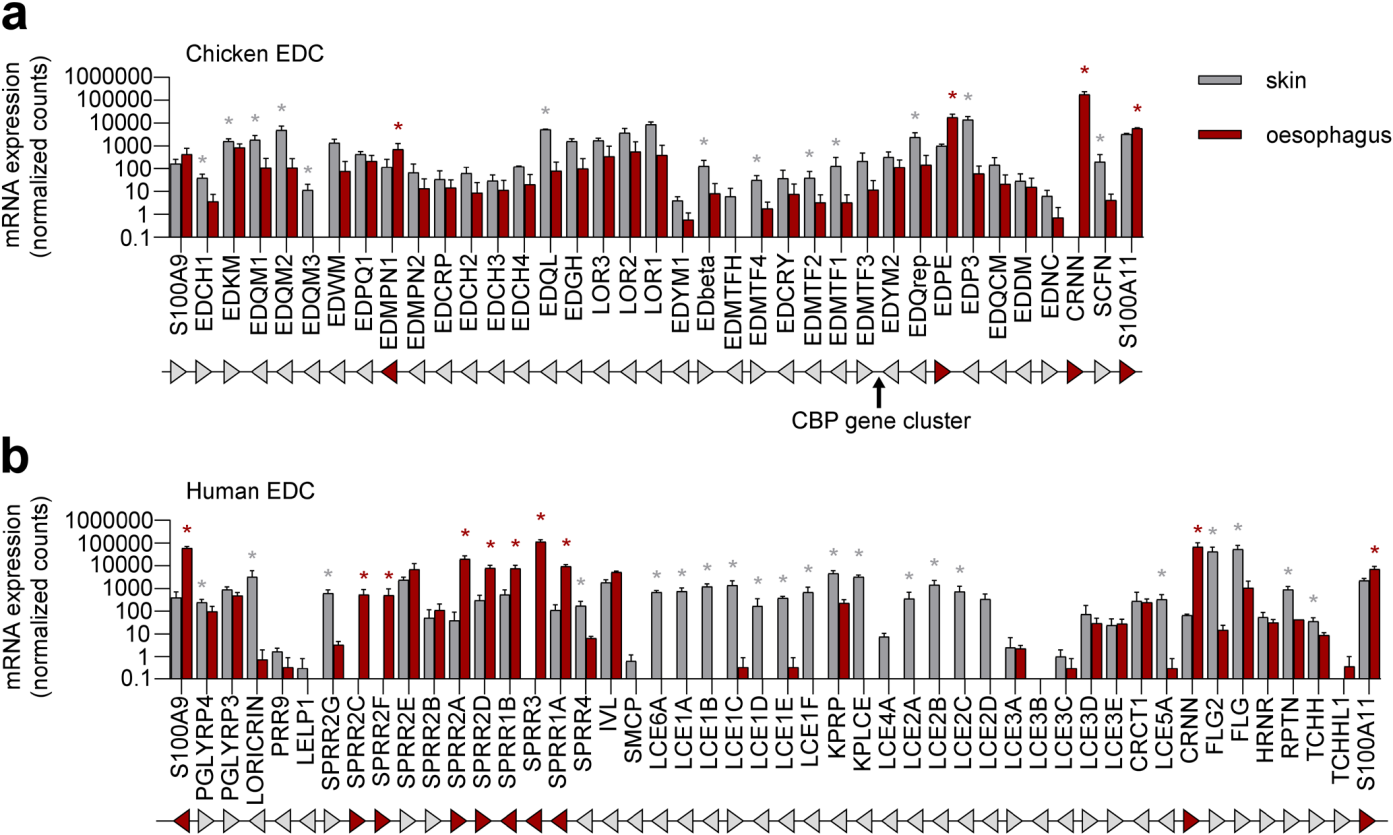
Expression of EDC genes in the skin and oesophagus of chickens and humans. The expression levels of EDC genes in the skin (grey bars) and the oesophagus (red bars) of the chicken (a) and humans (b) are shown in bar charts using a logarithmic scale. Bars represent the mean of the normalised read counts in the RNA‐seq analysis of three biological replicates. Error bars indicate standard deviations. Asterisks indicate significant differences (adjusted *p*‐value < 0.05), with grey asterisks highlighting enrichment in the skin and red asterisks highlighting enrichment in the oesophagus. The genes are sorted according to their arrangement in the EDC, which is schematically depicted below each bar chart. Genes are represented by triangles pointing in the direction of transcription. Red triangles highlight genes predominantly expressed in the oesophagus. Note that the general structure of the EDC is conserved between chickens and humans, whereas homology is limited at the level of individual genes [30].

The comparison of chicken skin and oesophagus transcriptomes showed that most of the EDC genes are expressed in both organs (Figure 2a). Genes encoding epidermal differentiation protein rich in glutamine repeats (EDQrep) [29, 30], the S100 fused‐type protein *scaffoldin* (*SCFN*), epidermal differentiation protein rich in proline 3 (*EDP3*) and ten other canonical EDC proteins, are enriched in the skin (Figure 2a). By contrast, three chicken EDC genes, namely *CRNN*, *EDPE* and *EDMPN1*, are enriched in the oesophagus (Figure 2a).

Like its homologues in the chicken, most of the human EDC genes are expressed in both the skin and the oesophagus (Figure 2a). *LORICRIN*, *FLG*, *FLG2* and the majority of *LCE*s are predominantly expressed in the skin, whereas many *SPRR* genes and *CRNN* are expressed at higher levels in the oesophagus (Figure 2a). Notably, the oesophagus‐enriched EDC genes are predominantly expressed in late differentiated suprabasal cells of the human oesophagus (Table S8) [23]. Together, these data demonstrate that (i) the EDCs of both a non‐mammalian vertebrate, the chicken and the human EDC contain genes that are expressed in the oesophagus, and (ii) some of these EDC genes are expressed at higher levels in the oesophagus than in the skin.

## 5 Discussion

The results of this study show that the EDC gene cluster is not only an evolutionarily ancient determinant of the barrier function of the skin, but also a conserved regulator of the oesophagus. The demonstration of EDC gene expression in the oesophagus of the chicken indicates that the dual role of the EDC has evolved in a common ancestor of humans and chickens, which—according to the current estimates of molecular phylogenetics [52]—has lived at least 310 million years ago. From this, we conclude that the gene cluster's name, EDC, overemphasises its role in the epidermis. Apparently, the EDC contributed to epithelial cell differentiation in the oesophagus already in the earliest phase of the evolution of amniotes, and this function has been conserved.

The relative expression levels of EDC genes in the skin and oesophagus differ substantially between humans and mice (Tables S4 and S5), which is in line with differences in the histology of the oesophagus. For example, most LCE genes are expressed specifically in the skin in humans (Figure 2b), whereas similar expression levels are found in murine skin and oesophagus (Table S5). It is likely that adaptations of the oesophagus in subgroups of both mammals and birds—possibly depending on the food and the lineage‐specific mode of its processing by chewing or swallowing whole or in chunks—have led to the diversifying evolution of gene expression in the oesophagus. To substantiate hypotheses about evolutionary trends, further species need to be studied in addition to the model species investigated here.

Although our analysis was focused on the EDC, our data also reveal additional sets of genes with expression patterns that are conserved across species (Tables S6 and S7). Three transcription factors of the forkhead‐box family (FOXA1, FOXF1 and FOXF2) and PAX9 show conservation of enriched expression in the oesophagus. Furthermore, PADI1, TGM1, GJB2 and the antimicrobial protein CYSRT1 [53] are consistently linked to the oesophagus (Table S6). *MAL* and *RHCG*, which are upregulated during the differentiation of human oesophageal epithelial cells (Table S8), are expressed in an oesophagus‐enriched manner in both chickens and humans (Table S7), but not in the mouse (Table S5).

Our study furthers the understanding of the evolutionary basis for commonalities and differences between the epidermis and other stratified epithelia such as the oesophageal epithelium. The dual function of the EDC in the skin and the oesophagus may have medical implications. According to the current state of knowledge, distinct EDC genes are dysregulated in skin diseases [4, 5, 9] and diseases of the oesophagus [22, 24, 25, 28]. Examples include defects of filaggrin in atopic dermatitis [4, 5, 9] and the downregulation of cornulin and SPRRs in oesophageal epithelial cancers [22, 24, 25, 28]. Given the shared expression of many EDC genes, it will be interesting to explore whether some pathological defects of the skin and the oesophagus share an aberrant expression of specific EDC genes.

## Author Contributions

A.P.S. and L.E. conceived the study; A.P.S., J.L., B.G. and V.M. performed investigations; A.P.S., J.L., V.M. and L.E. analysed data; A.P.S. and L.E. wrote the first draft of the manuscript; A.P.S., J.L., V.M., C.H., W.S. and L.E. revised the manuscript and approved the final version of the manuscript.

## Funding

This work was supported by the Austrian Science Fund.

## Ethics Statement

Animal procedures were approved by the Ethics and Animal Welfare Committee of the University of Veterinary Medicine, Vienna, Austria and the Austrian Federal Ministry of Education, Science and Research (licence number BMBWF GZ: 2021 0.881.824) and by the Ethics Review Committee for Animal Experimentation of the Medical University of Vienna, Austria and the Federal Ministry of Science, Research and Economy, Austria (GZ66.009/0255‐II/3b/2013). All methods were performed in accordance with the relevant guidelines and regulations. This study is reported in accordance with ARRIVE guidelines.

## Conflicts of Interest

The authors declare no conflicts of interest.

## Supporting information


**Table S1:** RNA‐seq data used for the study.
**Table S2:** Top 200 genes expressed in the chicken oesophagus (ranked by expression level).
**Table S6:** Oesophagus‐enriched gene expression which is conserved in human, mouse and chicken.
**Table S7:** Oesophagus‐enriched gene expression that is conserved in chicken and human.
**Table S8:** Categorisation of human oesophageal epithelial differentiation markers (based on 23, Late suprabasal cells).
**Figure S1:** Expression of EDC genes encoding corneous beta proteins (CBPs) in the skin and oesophagus of chickens.

**Table S3:** Expression levels (normalised read counts) of genes significantly (*p*_adj_ < 0.05) enriched in the oesophagus (log2FC > 1.5) or the skin (log2FC < −1.5) of the chicken.

**Table S4:** Expression levels (normalised read counts) of genes significantly (*p*_adj_ < 0.05) enriched in the human oesophagus (log2FC > 1.5) or the skin (log2FC < −1.5).

**Table S5:** Expression levels (normalised read counts) of genes significantly (*p*_adj_ < 0.05) enriched in the oesophagus (log2FC > 1.5) or the skin (log2FC < −1.5) of the mouse.

## Data Availability

The datasets generated and/or analysed during the current study are available in the NCBI GenBank repository, accession number PRJNA1222808 (chicken oesophagus, 3 replicates); accession number PRJNA1222777 (chicken oesophagus containing high level of muscle tissue); https://www.ncbi.nlm.nih.gov/datasets/genome/GCF_000002315.4/, GCA_000002315.3 (chicken reference genome); https://www.ncbi.nlm.nih.gov/datasets/genome/GCF_000001405.40/, GCF_000001405.40 (human reference genome); https://www.ncbi.nlm.nih.gov/datasets/genome/GCF_000001635.27/, GCF_000001635.27. Other datasets generated and/or analysed during the current study are available in the Zenodo repository, https://doi.org/10.5281/zenodo.14794868 (chicken reference genome used for read quantification) and https://doi.org/10.5281/zenodo.14930419 (complete and unfiltered RNA‐seq results for chicken, human and mouse).
