# Supplementary material for: Comparative Transcriptomics Reveals a Dual Role of the Epidermal Differentiation Complex in the Skin and the Oesophagus
Source: Exp Dermatol. 2025 Nov 30;34(12):e70181. doi: 10.1111/exd.70181 (PMC12665336; doi:10.1111/exd.70181)
Supplement: Supplementary file 1 — Table S1: RNA‐seq data used for the study. Table S2: Top 200 genes expressed in the chicken oesophagus (ranked by expression level). Table S6: Oesophagus‐enriched gene expression which is conserved in human, mouse and chicken. Table S7: Oesophagus‐enriched gene expression that is conserved in chicken and human. Table S8: Categorisation of human oesophageal epithelial differentiation markers (based on 23, Late suprabasal cells). Figure S1: Expression of EDC genes encoding corneous beta proteins (CBPs) in the skin and oesophagus of chickens. [file EXD-34-e70181-s004.docx]

**Supplementary Data: Supplementary Tables S1, S2, S6-S8 and Supplementary Figure S1**

**Comparative transcriptomics reveals a dual role of the epidermal differentiation complex in the skin and the esophagus**

Attila Placido Sachslehner, Julia Lachner, Veronika Mlitz, Bahar Golabi, Claudia Hess, Wolfgang Sipos, Leopold Eckhart

**Content**

Supplementary methods

Supplementary Tables S1, S2, S6-S8

Supplementary Figure S1

**Supplementary methods**

**Histology**

For histological analysis, esophagus samples were incubated in 7.5% neutral-buffered formalin (Morphisto, Offenbach am Main, Germany) overnight. Subsequently, the samples were embedded in paraffin. Sections were prepared with a HM335E microtome (Zeiss, Germany) at a thickness of 5 µm and collected in a waterbath set to 42°C. The sections were subjected to hematoxylin and eosin (H&E) staining and photographed with an Olympus BX63 light microscope equipped with a UC-90 camera and the software cellSens Dimension (version: 2.3.18987.0).

**RNA-sequencing**

RNA sequencing was performed at the Core Facility Genomics, Medical University of Vienna, a member of Vienna Life-Science Instruments (VLSI). Sequencing libraries from total RNA of the samples were prepared using the NEBNext Poly(A) mRNA Magnetic Isolation Module and the NEBNext UltraExpress RNA Library Prep Kit for Illumina with Unique Dual Indices (UDIs) according to manufacturer's protocols (New England Biolabs). Libraries were tested for correct insert size using a High Sensitivity DNA Kit on a Bioanalyzer 2100 (Agilent) and quantified using Qubit dsDNA HS Assay (Invitrogen). Libraries were sequenced on a P4 flowcell of a NextSeq2000 instrument in 1x50 bp single-end sequencing mode. On average, 22 million reads per sample were generated.

**Quantification of human and mouse RNA-seq reads**

RNA-seq reads derived from esophagus and skin samples from human and mouse (Supplementary Table S1) were downloaded and converted to fastq files from NCBI GenBank with the prefetch and fastq-dump package from SRA Toolkit respectively (version: 3.1.1, https://github.com/ncbi/sra-tools, last accessed on January 7, 2025) and subjected to quality -control with FastQC (version 0.12.1, https://www.bioinformatics.babraham.ac.uk/projects/fastqc/, last accessed on January 7, 2025). Sequencing adapters of the mouse skin read files (PRJNA1039300) were removed with Trimmomatic (version: 0.39). The reads were quantified with respect to the actual reference genomes of human (NCBI GenBank accession: GCF_000001405.40) and mouse (NCBI GenBank accession: GCF_000001635.27) respectively. Differential gene expression analysis was performed as described for the chicken.

| **Supplementary Table S1. RNA-seq data used for the study** | | | | | |
| --- | --- | --- | --- | --- | --- |
| Species | Species (scientific name) | Tissue | Bioproject (GenBank) | SRA accession number | Transcriptome size [Gb] |
| Chicken | *Gallus gallus* | esophagus 1 | PRJNA1222808 | SRR32319409 | 1.2 |
| Chicken | *Gallus gallus* | esophagus 2 | PRJNA1222808 | SRR32319408 | 1.1 |
| Chicken | *Gallus gallus* | esophagus 3 | PRJNA1222808 | SRR32319407 | 4.8 |
| Chicken | *Gallus gallus* | skin 1 | PRJNA1021778 | SRX21911437 | 4.7 |
| Chicken | *Gallus gallus* | skin 2 | PRJNA1021778 | SRX21911438 | 5.2 |
| Chicken | *Gallus gallus* | skin 3 | PRJNA1021778 | SRX21911440 | 6.4 |
| Human | *Homo sapiens* | esophagus 1 | PRJEB4337 | ERX288521 | 3.2 |
| Human | *Homo sapiens* | esophagus 2 | PRJEB4337 | ERX288503 | 3.6 |
| Human | *Homo sapiens* | esophagus 3 | PRJEB4337 | ERX288510 | 2.7 |
| Human | *Homo sapiens* | skin 1 | PRJEB4337 | ERX288474 | 2.7 |
| Human | *Homo sapiens* | skin 2 | PRJEB4337 | ERX288593 | 2.9 |
| Human | *Homo sapiens* | skin 3 | PRJEB4337 | ERX288485 | 3.3 |
| Mouse | *Mus musculus* | esophagus 1 | PRJEB22693 | ERX2187477 | 1.7 |
| Mouse | *Mus musculus* | esophagus 2 | PRJEB22693 | ERX2187476 | 1.2 |
| Mouse | *Mus musculus* | esophagus 3 | PRJEB22693 | ERX2187475 | 1.2 |
| Mouse | *Mus musculus* | skin 1 | PRJNA1039300 | SRX22490455 | 3.8 |
| Mouse | *Mus musculus* | skin 2 | PRJNA1039300 | SRX22490456 | 4.0 |
| Mouse | *Mus musculus* | skin 3 | PRJNA1039300 | SRX22490457 | 3.9 |

| **Supplementary Table S2. Top 200 genes expressed in the chicken esophagus (ranked by expression level)** | | | | | | | |
| --- | --- | --- | --- | --- | --- | --- | --- |
| Gene | Esophagus 1 | Esophagus 2 | Esophagus 3 | Mean | SD | CV | Alternative gene name |
| *KRT15* | 807796 | 772197 | 569428 | 716474 | 104988 | 15% |  |
| *KRT78L1* | 755143 | 680862 | 572046 | 669350 | 75191 | 11% |  |
| *COL3A1* | 484477 | 296607 | 395880 | 392321 | 76739 | 20% |  |
| *ENSGALG00000031496* | 234957 | 231101 | 232542 | 232867 | 1591 | 1% | *SPINK5-like* |
| *MT-CO1* | 204567 | 215826 | 223964 | 214786 | 7953 | 4% |  |
| *CRNN* | 239649 | 172422 | 131260 | 181111 | 44674 | 25% |  |
| *ACTG2* | 130429 | 177857 | 232670 | 180319 | 41776 | 23% |  |
| *MYH11* | 154279 | 190172 | 159814 | 168088 | 15778 | 9% |  |
| *COL1A2* | 226879 | 123022 | 142614 | 164172 | 45057 | 27% |  |
| *ACTB* | 130629 | 128996 | 109036 | 122887 | 9817 | 8% |  |
| *SPARC* | 168397 | 77667 | 79640 | 108568 | 42313 | 39% |  |
| *EEF1A1* | 116241 | 106292 | 90475 | 104336 | 10609 | 10% |  |
| *ATP6* | 98800 | 98820 | 88304 | 95308 | 4953 | 5% |  |
| *TAGLN* | 84705 | 106157 | 81813 | 90891 | 10859 | 12% |  |
| *COX3* | 95372 | 91046 | 85863 | 90760 | 3888 | 4% |  |
| *MYLK* | 78923 | 87765 | 89039 | 85242 | 4498 | 5% |  |
| *ENSGALG00000003521* | 76249 | 88436 | 90849 | 85178 | 6390 | 8% | *TPM1* |
| *ENSGALG00000031223* | 54104 | 78086 | 118040 | 83410 | 26372 | 32% |  |
| *MT-CYB* | 73975 | 77905 | 87557 | 79812 | 5707 | 7% |  |
| *DES* | 75912 | 91160 | 68125 | 78399 | 9567 | 12% |  |
| *MT-CO2* | 75788 | 78521 | 68337 | 74215 | 4304 | 6% |  |
| *TPM2* | 85072 | 70093 | 62273 | 72479 | 9459 | 13% |  |
| *ND5* | 64896 | 58463 | 83394 | 68918 | 10568 | 15% |  |
| *EMP1* | 69588 | 80237 | 54148 | 67991 | 10710 | 16% |  |
| *ANXA1* | 94828 | 56091 | 49304 | 66741 | 20053 | 30% |  |
| *ENSGALG00000036073* | 108259 | 38918 | 39690 | 62289 | 32507 | 52% | *COL1A1* |
| *ENSGALG00000028749* | 70508 | 60402 | 43358 | 58090 | 11204 | 19% | *ACTG1* |
| *ENSGALG00000028567* | 51917 | 65294 | 46732 | 54647 | 7820 | 14% | *MYL9* |
| *MT-ND4* | 49416 | 47874 | 62034 | 53108 | 6343 | 12% |  |
| *ENSGALG00000013072* | 29090 | 29812 | 100363 | 53088 | 33429 | 63% | *AKR1B10* |
| *ANXA2* | 60513 | 46002 | 52680 | 53065 | 5931 | 11% |  |
| *ND1* | 50417 | 46151 | 58156 | 51575 | 4969 | 10% |  |
| *COL1A1* | 68779 | 29044 | 52884 | 50236 | 16329 | 33% |  |
| *CSRP1* | 48329 | 58934 | 37305 | 48189 | 8831 | 18% |  |
| *MYL6* | 43241 | 58084 | 36068 | 45798 | 9168 | 20% |  |
| *COL6A2* | 48241 | 36567 | 40679 | 41829 | 4835 | 12% |  |
| *ENSGALG00000035155* | 32153 | 41553 | 44920 | 39542 | 5403 | 14% |  |
| *CKB* | 44494 | 45352 | 28080 | 39309 | 7948 | 20% |  |
| *ENSGALG00000004509* | 40681 | 49549 | 26873 | 39034 | 9330 | 24% | *LOC101747587* |
| *ENSGALG00000007220* | 42231 | 38448 | 33035 | 37905 | 3774 | 10% | *FTH1* |
| *MT-ND2* | 38937 | 38621 | 35030 | 37529 | 1772 | 5% |  |
| *GAPDH* | 38438 | 40654 | 28941 | 36011 | 5080 | 14% |  |
| *ENSGALG00000029182* | 45171 | 25074 | 36858 | 35701 | 8245 | 23% |  |
| *CSTA* | 29901 | 35019 | 41373 | 35431 | 4692 | 13% |  |
| *CALD1* | 27774 | 42521 | 33747 | 34681 | 6057 | 17% |  |
| *ENSGALG00000044239* | 1880 | 2319 | 93134 | 32445 | 42915 | 132% |  |
| *CALM* | 36041 | 33566 | 27243 | 32283 | 3704 | 11% |  |
| *EEF2* | 30715 | 32231 | 31906 | 31617 | 652 | 2% |  |
| *PABPC1* | 34078 | 33229 | 25558 | 30955 | 3832 | 12% |  |
| *GSN* | 32170 | 39332 | 20572 | 30691 | 7730 | 25% |  |
| *KRT5L1* | 26921 | 32808 | 32092 | 30607 | 2623 | 9% |  |
| *COL6A1* | 33545 | 24707 | 30638 | 29630 | 3678 | 12% |  |
| *DCN* | 38249 | 18339 | 24141 | 26910 | 8361 | 31% |  |
| *EIF4G2* | 26390 | 23947 | 23971 | 24769 | 1146 | 5% |  |
| *Wpkci-8* | 50 | 48190 | 25221 | 24487 | 19660 | 80% |  |
| *RPS3A* | 26464 | 27319 | 18926 | 24237 | 3771 | 16% |  |
| *COL6A3* | 22623 | 16187 | 32700 | 23837 | 6796 | 29% |  |
| *MGP* | 27687 | 22864 | 18640 | 23063 | 3696 | 16% |  |
| *DSTN* | 23352 | 22047 | 22973 | 22791 | 548 | 2% |  |
| *RPLP0* | 22364 | 23148 | 21828 | 22446 | 542 | 2% |  |
| *MAL* | 20162 | 20022 | 26086 | 22090 | 2826 | 13% |  |
| *ENSGALG00000008684* | 22609 | 23123 | 19185 | 21639 | 1748 | 8% | *EIF4A2* |
| *ENSGALG00000040736* | 14655 | 14325 | 35263 | 21414 | 9793 | 46% |  |
| *KRT8* | 20940 | 23454 | 19512 | 21302 | 1630 | 8% |  |
| *ENSGALG00000032940* | 16818 | 22503 | 24264 | 21195 | 3177 | 15% |  |
| *RPL4* | 20965 | 21568 | 20633 | 21055 | 387 | 2% |  |
| *ENSGALG00000015704* | 29085 | 16978 | 16458 | 20841 | 5834 | 28% | *TXN* |
| *TPT1* | 21753 | 22017 | 17897 | 20556 | 1883 | 9% |  |
| *HSPA2* | 19995 | 20860 | 19903 | 20253 | 431 | 2% |  |
| *FN1* | 19099 | 14426 | 26670 | 20065 | 5045 | 25% |  |
| *DSP* | 16266 | 20789 | 22043 | 19699 | 2481 | 13% |  |
| *CBP64-K* | 16702 | 11207 | 31096 | 19668 | 8386 | 43% |  |
| *FSTL1* | 27636 | 15433 | 15543 | 19537 | 5727 | 29% |  |
| *TUBA1A* | 24346 | 19705 | 13594 | 19215 | 4403 | 23% |  |
| *PPL* | 16329 | 21474 | 19282 | 19028 | 2108 | 11% |  |
| *ENSGALG00000016691* | 18860 | 16923 | 20612 | 18798 | 1507 | 8% | *SLC25A6* |
| *CAVIN1* | 17394 | 18211 | 20455 | 18686 | 1294 | 7% |  |
| *COL5A1* | 23414 | 13743 | 18377 | 18511 | 3949 | 21% |  |
| *ENSGALG00000036956* | 17977 | 16257 | 21009 | 18414 | 1964 | 11% |  |
| *ENSGALG00000002377* | 18408 | 18422 | 17988 | 18273 | 201 | 1% | *ENO1* |
| *EDPE-EDC* | 21749 | 11095 | 21851 | 18232 | 5046 | 28% |  |
| *RPS2* | 18695 | 17002 | 18587 | 18095 | 774 | 4% |  |
| *YBX1* | 17608 | 17241 | 19324 | 18058 | 908 | 5% |  |
| *FABP3* | 18584 | 20395 | 15156 | 18045 | 2173 | 12% |  |
| *GNB2L1* | 19541 | 19177 | 14903 | 17874 | 2106 | 12% |  |
| *ITM2A* | 20575 | 17472 | 15554 | 17867 | 2069 | 12% |  |
| *RPL5* | 17844 | 19041 | 16668 | 17851 | 969 | 5% |  |
| *RPS6* | 27156 | 14648 | 11627 | 17810 | 6723 | 38% |  |
| *RHOA* | 20695 | 17022 | 14816 | 17511 | 2425 | 14% |  |
| *S100A6* | 15569 | 20275 | 16039 | 17294 | 2116 | 12% |  |
| *TGM1* | 18520 | 18242 | 14957 | 17240 | 1618 | 9% |  |
| *RPS8* | 19271 | 19451 | 12644 | 17122 | 3167 | 18% |  |
| *ITGB1* | 16006 | 16386 | 18899 | 17097 | 1284 | 8% |  |
| *RPL3* | 17129 | 17487 | 16472 | 17029 | 420 | 2% |  |
| *ENSGALG00000026383* | 23632 | 15978 | 11436 | 17015 | 5033 | 30% | *TMSB4X* |
| *CNN1* | 11182 | 17012 | 22456 | 16883 | 4603 | 27% |  |
| *RPSA* | 16991 | 18413 | 14758 | 16721 | 1504 | 9% |  |
| *JUP* | 16081 | 17134 | 16661 | 16625 | 431 | 3% |  |
| *RPL8* | 18159 | 17728 | 13798 | 16562 | 1962 | 12% |  |
| *ENSGALG00000044418* | 23892 | 19108 | 6057 | 16352 | 7538 | 46% | *MUC5L* |
| *ENSGALG00000043598* | 11411 | 13255 | 24127 | 16264 | 5611 | 34% |  |
| *CLDN1* | 14199 | 17969 | 16508 | 16225 | 1552 | 10% |  |
| *ALDOC* | 15948 | 16007 | 15688 | 15881 | 139 | 1% |  |
| *CD81* | 18154 | 15137 | 14196 | 15829 | 1688 | 11% |  |
| *ENSGALG00000032220* | 33195 | 8839 | 4972 | 15668 | 12493 | 80% | *ELN* |
| *SYNM* | 14647 | 19669 | 12551 | 15622 | 2986 | 19% |  |
| *HSPG2* | 15150 | 16446 | 14628 | 15408 | 764 | 5% |  |
| *ACTN1* | 12344 | 15904 | 17962 | 15403 | 2321 | 15% |  |
| *HSP90AA1* | 19169 | 16835 | 10122 | 15375 | 3835 | 25% |  |
| *ENSGALG00000004725* | 15986 | 16504 | 13295 | 15261 | 1407 | 9% | *ALDH2* |
| *COL5A2* | 17120 | 11247 | 17175 | 15181 | 2782 | 18% |  |
| *PRDX1* | 15092 | 15552 | 14203 | 14949 | 560 | 4% |  |
| *ENSGALG00000005956* | 12061 | 14343 | 17842 | 14749 | 2378 | 16% | *ANXA8L1* |
| *P4HB* | 15121 | 12098 | 16656 | 14625 | 1894 | 13% |  |
| *RPL6* | 15286 | 15267 | 12327 | 14293 | 1391 | 10% |  |
| *PPP1CB* | 13714 | 13472 | 15000 | 14062 | 671 | 5% |  |
| *ENSGALG00000011865* | 13260 | 15704 | 13129 | 14031 | 1184 | 8% | *SMTN* |
| *SERPINH1* | 18354 | 12497 | 11149 | 14000 | 3127 | 22% |  |
| *ATP5B* | 13564 | 14331 | 13840 | 13912 | 317 | 2% |  |
| *ENSGALG00000028600* | 13773 | 13501 | 13869 | 13715 | 156 | 1% | *PPIA* |
| *ENSGALG00000001250* | 14014 | 13504 | 13310 | 13609 | 297 | 2% | *APLP2* |
| *OAZ1* | 16487 | 14107 | 9993 | 13529 | 2683 | 20% |  |
| *ENSGALG00000000081* | 10674 | 11884 | 17632 | 13397 | 3035 | 23% | *IL4I1* |
| *ENSGALG00000005349* | 11032 | 11278 | 17766 | 13359 | 3118 | 23% | *MSLN* |
| *ENSGALG00000015702* | 8863 | 5933 | 25236 | 13344 | 8493 | 64% | *PTGR1* |
| *ENSGALG00000006343* | 9927 | 10107 | 19857 | 13297 | 4639 | 35% | *ACTA2* |
| *UPK1B* | 12137 | 14507 | 13211 | 13285 | 969 | 7% |  |
| *ENSGALG00000001885* | 13942 | 15473 | 10361 | 13259 | 2142 | 16% | *UPK3BL* |
| *RPS4* | 12835 | 14033 | 12645 | 13171 | 614 | 5% |  |
| *SERBP1* | 14412 | 14125 | 10942 | 13160 | 1573 | 12% |  |
| *ENSGALG00000039470* | 14566 | 13282 | 11087 | 12978 | 1436 | 11% |  |
| *ENSGALG00000023925* | 13464 | 16327 | 8907 | 12899 | 3055 | 24% | *CYP2C18* |
| *EPCAM* | 14375 | 13634 | 10641 | 12883 | 1614 | 13% |  |
| *RPL7A* | 13289 | 13205 | 12007 | 12834 | 585 | 5% |  |
| *HSPA8* | 16373 | 11799 | 10175 | 12782 | 2624 | 21% |  |
| *DDX5* | 12589 | 10920 | 14807 | 12772 | 1592 | 12% |  |
| *COL4A1* | 9565 | 12098 | 16532 | 12732 | 2879 | 23% |  |
| *GPX3* | 14314 | 14434 | 9066 | 12605 | 2503 | 20% |  |
| *RPL7* | 12282 | 13362 | 12169 | 12604 | 538 | 4% |  |
| *RPL15* | 12589 | 13193 | 11517 | 12433 | 693 | 6% |  |
| *SYNPO2* | 11119 | 13521 | 12135 | 12259 | 985 | 8% |  |
| *RPL19* | 14106 | 13743 | 8436 | 12095 | 2591 | 21% |  |
| *ENSGALG00000042435* | 11168 | 15988 | 8772 | 11976 | 3001 | 25% |  |
| *HSPB1* | 9947 | 13612 | 12209 | 11923 | 1510 | 13% |  |
| *CNBP* | 13226 | 12125 | 10219 | 11857 | 1242 | 10% |  |
| *RPS24* | 11665 | 13289 | 10587 | 11847 | 1110 | 9% |  |
| *APOA1* | 21807 | 6908 | 6794 | 11837 | 7051 | 60% |  |
| *ENSGALG00000028520* | 17383 | 12243 | 5880 | 11836 | 4705 | 40% | *CST3* |
| *ANXA5* | 13157 | 10909 | 10923 | 11663 | 1057 | 9% |  |
| *RPS12* | 10940 | 13187 | 10058 | 11395 | 1317 | 12% |  |
| *MYO18A* | 10283 | 12835 | 11051 | 11390 | 1069 | 9% |  |
| *YWHAQ* | 12111 | 10433 | 11549 | 11364 | 697 | 6% |  |
| *RPS3* | 11503 | 11839 | 10658 | 11333 | 497 | 4% |  |
| *VIM* | 14944 | 9234 | 9798 | 11325 | 2569 | 23% |  |
| *ENSGALG00000016231* | 11253 | 11751 | 10923 | 11309 | 340 | 3% | *DDX3X* |
| *CIRBP* | 10663 | 11789 | 11174 | 11209 | 460 | 4% |  |
| *ENSGALG00000011930* | 8316 | 12153 | 12327 | 10932 | 1851 | 17% | *OVSTL* |
| *RPL9* | 11007 | 11959 | 9810 | 10925 | 879 | 8% |  |
| *PKM* | 11615 | 10586 | 10543 | 10915 | 495 | 5% |  |
| *RPS19* | 12783 | 12265 | 7528 | 10859 | 2365 | 22% |  |
| *RPL27* | 11786 | 12543 | 8200 | 10843 | 1894 | 17% |  |
| *ENSGALG00000046509* | 4704 | 6887 | 20411 | 10668 | 6947 | 65% |  |
| *HDLBP* | 10260 | 10485 | 10928 | 10558 | 278 | 3% |  |
| *ENSGALG00000039810* | 62 | 82 | 31387 | 10510 | 14762 | 140% |  |
| *PIGR* | 12950 | 12101 | 6109 | 10387 | 3045 | 29% |  |
| *FHL2* | 11483 | 9437 | 10238 | 10386 | 842 | 8% |  |
| *EEF1B2* | 10849 | 10880 | 9293 | 10341 | 741 | 7% |  |
| *PSCA* | 5856 | 13049 | 11903 | 10269 | 3156 | 31% |  |
| *NMRAL1* | 12690 | 6774 | 10984 | 10149 | 2486 | 24% |  |
| *PGD* | 10312 | 9949 | 10165 | 10142 | 149 | 1% |  |
| *HOPX* | 11304 | 14606 | 4491 | 10134 | 4211 | 42% |  |
| *ENSGALG00000000866* | 11379 | 9249 | 9662 | 10097 | 922 | 9% | *SELENBP1* |
| *ENSGALG00000033026* | 7235 | 8434 | 14544 | 10071 | 3200 | 32% |  |
| *RPS16* | 11617 | 9571 | 8893 | 10027 | 1158 | 12% |  |
| *ENSGALG00000041031* | 12838 | 7242 | 9847 | 9976 | 2287 | 23% | *TNX* |
| *RPL13* | 10395 | 11022 | 8434 | 9950 | 1103 | 11% |  |
| *GNB1* | 10598 | 9384 | 9656 | 9879 | 520 | 5% |  |
| *FBN1* | 15705 | 6467 | 7306 | 9826 | 4171 | 42% |  |
| *ENSGALG00000030736* | 246 | 252 | 28877 | 9791 | 13495 | 138% |  |
| *RPS10-NUDT3* | 10199 | 10614 | 8558 | 9790 | 888 | 9% |  |
| *ITM2B* | 11672 | 10115 | 7460 | 9749 | 1739 | 18% |  |
| *TPM3* | 10103 | 9669 | 9455 | 9742 | 270 | 3% |  |
| *ENSGALG00000030120* | 10229 | 9103 | 9741 | 9691 | 461 | 5% | *ITGB1BP3* |
| *SFN* | 9651 | 10509 | 8903 | 9688 | 656 | 7% |  |
| *ADIPOQ* | 11402 | 9918 | 7704 | 9675 | 1520 | 16% |  |
| *PYGB* | 9647 | 11657 | 7703 | 9669 | 1614 | 17% |  |
| *ATP2B4* | 7461 | 11532 | 9801 | 9598 | 1668 | 17% |  |
| *POF1B* | 7863 | 9708 | 11186 | 9586 | 1359 | 14% |  |
| *RPL21* | 9605 | 9580 | 9387 | 9524 | 98 | 1% |  |
| *ATF4* | 10560 | 9405 | 8444 | 9469 | 865 | 9% |  |
| *ATP1A1* | 9653 | 9722 | 8975 | 9450 | 337 | 4% |  |
| *ENSGALG00000042353* | 63 | 81 | 27996 | 9380 | 13163 | 140% |  |
| *ENSGALG00000045810* | 0 | 0 | 28123 | 9374 | 13257 | 141% |  |
| *RPS14* | 10183 | 10321 | 7482 | 9328 | 1307 | 14% |  |
| *ENSGALG00000021450* | 5676 | 8060 | 14247 | 9327 | 3612 | 39% |  |
| *NPM1* | 9444 | 8741 | 9693 | 9292 | 403 | 4% |  |
| *CDH1* | 8528 | 10207 | 9084 | 9273 | 698 | 8% |  |
| *PDLIM7* | 7763 | 9942 | 9971 | 9225 | 1034 | 11% |  |
| *YWHAG* | 8805 | 9331 | 9501 | 9212 | 296 | 3% |  |
| *NCL* | 9099 | 8820 | 9687 | 9202 | 361 | 4% |  |
| Notes: CV, coefficient of variation; SD; standard deviation | | | | | | |  |

**Note: Supplementary Tables S3, S4 and S5 are provided as separate files in Excel format.**

| **Supplementary Table S6. Esophagus-enriched gene expression which is conserved in human, mouse and chicken** | | | | | | | |  |
| --- | --- | --- | --- | --- | --- | --- | --- | --- |
| Gene | log2FC | log2FC | log2FC | Padj | Padj | Padj | Gene function | |
|  | chicken | human | mouse | chicken | human | mouse |  | |
| *fam3d* | 10.94 | 7.20 | 11.50 | 1.67E-13 | 1.43E-46 | 5.02E-08 | metabolism regulating signaling molecule | |
| *foxa1* | 10.26 | 8.80 | 10.90 | 3.25E-21 | 1.38E-08 | 1.19E-12 | transcription factor | |
| *foxf2* | 9.82 | 3.90 | 6.60 | 5.26E-06 | 5.60E-20 | 4.79E-16 | transcription factor | |
| *tbx5* | 9.21 | 3.70 | 6.10 | 2.45E-05 | 1.50E-11 | 4.56E-14 | transcription factor | |
| *psca* | 8.44 | 4.60 | 3.40 | 6.88E-73 | 6.42E-04 | 2.27E-05 | cell membrane glycoprotein | |
| *pax9* | 8.37 | 8.90 | 10.10 | 7.26E-32 | 2.87E-60 | 2.45E-75 | transcription factor | |
| *pitx1* | 8.23 | 7.80 | 6.70 | 2.44E-93 | 3.53E-10 | 1.79E-85 | transcription factor | |
| *prr15l* | 7.27 | 3.10 | 2.80 | 2.12E-03 | 1.47E-05 | 7.30E-04 | proline-rich protein 15-like | |
| *tmprss2* | 6.23 | 7.00 | 2.30 | 2.16E-20 | 1.14E-48 | 3.04E-08 | transmembrane protease | |
| *sox2* | 6.20 | 5.40 | 5.70 | 2.63E-43 | 7.70E-28 | 3.82E-54 | transcription factor | |
| *tcf21* | 5.93 | 10.50 | 9.90 | 4.89E-35 | 2.08E-06 | 3.68E-06 | transcription factor | |
| *cysrt1* | 5.84 | 2.12 | 2.77 | 5.26E-49 | 1.14E-02 | 6.72E-17 | antimicrobial protein | |
| *elf3* | 5.58 | 6.40 | 4.50 | 3.28E-54 | 2.19E-84 | 1.66E-18 | transcription factor | |
| *foxf1* | 5.42 | 6.10 | 6.70 | 2.38E-30 | 1.69E-23 | 1.47E-28 | transcription factor | |
| *gjb2* | 4.88 | 4.16 | 5.63 | 1.04E-31 | 2.87E-18 | 1.48E-60 | gap junction protein (connexin) | |
| *tgm1* | 4.86 | 4.10 | 2.10 | 1.48E-120 | 2.19E-39 | 1.51E-08 | transglutaminase | |
| *gpx2* | 4.81 | 2.50 | 7.00 | 2.32E-17 | 2.63E-12 | 1.37E-114 | protection against oxidative stress | |
| *casq2* | 4.70 | 7.10 | 2.30 | 2.68E-04 | 5.01E-41 | 2.29E-08 | calsequestrin 2, muscle function | |
| *rasef* | 4.35 | 2.70 | 4.00 | 2.95E-13 | 9.35E-07 | 1.93E-06 | Rab family GTPase, membrane traffic | |
| *ap1s3* | 3.82 | 2.10 | 2.10 | 2.93E-07 | 7.48E-06 | 1.30E-09 | adaptor related protein complex 1 | |
| *padi1* | 3.25 | 2.80 | 7.90 | 8.16E-09 | 1.17E-13 | 1.00E-56 | peptidyl arginine deimination (citrullination) | |
| *mgp* | 3.04 | 2.30 | 3.90 | 1.19E-18 | 3.11E-06 | 1.03E-12 | osteocalcin, matrix Gla family protein | |
| *vipr2* | 2.99 | 3.20 | 2.20 | 1.26E-09 | 9.82E-06 | 4.76E-06 | vasoactive intestinal peptide receptor | |
| *actg2* | 2.88 | 9.20 | 2.50 | 1.61E-26 | 1.27E-80 | 4.94E-05 | actin gamma 2, smooth muscle | |
| *eya2* | 2.81 | 3.60 | 3.10 | 2.44E-03 | 1.09E-19 | 4.96E-18 | transcriptional activator | |
| *nqo1* | 2.74 | 4.10 | 2.90 | 2.79E-15 | 1.03E-28 | 2.03E-15 | protection against oxidative stress | |
| *musk* | 2.66 | 3.60 | 2.40 | 1.54E-03 | 1.53E-09 | 5.67E-09 | muscle associated receptor tyrosine kinase | |
| *rarb* | 2.19 | 4.00 | 2.20 | 8.12E-15 | 3.16E-16 | 5.63E-24 | retinoic acid receptor | |

Notes: FC, fold change (esophagus versus skin); Padj, adjusted P-value.

| **Supplementary Table S7. Esophagus-enriched gene expression that is conserved in chicken and human.** | | | | |
| --- | --- | --- | --- | --- |
| Gene | log2FC | log2FC | Padj | Padj |
|  | chicken | human | chicken | human |
| *crnn* | 19.84 | 10.00 | 1.39E-22 | 3.78E-145 |
| *rhcg* | 12.0 | 11.4 | 1.49E-08 | 1.66E-97 |
| *krt18* | 11.7 | 4.0 | 3.75E-15 | 2.47E-17 |
| *fam3d* | 10.9 | 7.2 | 1.67E-13 | 1.43E-46 |
| *mal* | 10.3 | 7.9 | 1.29E-231 | 8.79E-215 |
| *foxa1* | 10.3 | 8.8 | 3.25E-21 | 1.38E-08 |
| *foxf2* | 9.8 | 3.9 | 5.26E-06 | 5.60E-20 |
| *tbx5* | 9.2 | 3.7 | 2.45E-05 | 1.50E-11 |
| *krt19* | 9.0 | 8.1 | 1.10E-130 | 6.53E-67 |
| *cldn10* | 8.9 | 6.5 | 9.89E-37 | 9.89E-06 |
| *psca* | 8.4 | 4.6 | 6.88E-73 | 6.42E-04 |
| *pax9* | 8.4 | 8.9 | 7.26E-32 | 2.87E-60 |
| *pitx1* | 8.2 | 7.8 | 2.44E-93 | 3.53E-10 |
| *tmprss4* | 7.6 | 3.4 | 3.85E-67 | 1.36E-13 |
| *prr15l* | 7.3 | 3.1 | 2.12E-03 | 1.47E-05 |
| *tspan1* | 7.2 | 2.3 | 6.13E-15 | 2.72E-02 |
| *tmprss2* | 6.2 | 7.0 | 2.16E-20 | 1.14E-48 |
| *sox2* | 6.2 | 5.4 | 2.63E-43 | 7.70E-28 |
| *chac1* | 6.0 | 6.3 | 3.67E-60 | 1.08E-15 |
| *tcf21* | 5.9 | 10.5 | 4.89E-35 | 2.08E-06 |
| *lypd2* | 5.8 | 2.9 | 2.59E-02 | 4.62E-10 |
| *elf3* | 5.6 | 6.4 | 3.28E-54 | 2.19E-84 |
| *foxf1* | 5.4 | 6.1 | 2.38E-30 | 1.69E-23 |
| *fam3b* | 5.3 | 6.1 | 1.11E-08 | 1.33E-30 |
| *itgb1bp2* | 5.3 | 3.1 | 2.34E-05 | 1.98E-04 |
| *aldh1a1* | 5.0 | 2.1 | 2.74E-26 | 4.71E-06 |
| *tgm1* | 4.9 | 4.1 | 1.48E-120 | 2.19E-39 |
| *gpx2* | 4.8 | 2.5 | 2.32E-17 | 2.63E-12 |
| *casq2* | 4.7 | 7.1 | 2.68E-04 | 5.01E-41 |
| *slc9a4* | 4.6 | 2.5 | 1.52E-02 | 4.59E-04 |
| *slc12a8* | 4.6 | 2.2 | 4.32E-11 | 7.50E-03 |
| *krt7* | 4.6 | 5.9 | 4.16E-31 | 2.41E-08 |
| *krt8* | 4.6 | 4.9 | 4.14E-69 | 2.31E-12 |
| *chrm3* | 4.4 | 7.7 | 1.13E-11 | 7.03E-24 |
| *rasef* | 4.4 | 2.7 | 2.95E-13 | 9.35E-07 |
| *gng4* | 4.3 | 5.1 | 8.74E-44 | 3.86E-16 |
| *clic5* | 4.2 | 2.1 | 2.28E-21 | 1.86E-02 |
| *alpk3* | 4.2 | 4.8 | 1.93E-04 | 5.43E-20 |
| *emp1* | 4.0 | 3.7 | 7.52E-53 | 2.24E-26 |
| *ap1s3* | 3.8 | 2.1 | 2.93E-07 | 7.48E-06 |
| *p2ry14* | 3.5 | 3.4 | 2.30E-27 | 6.03E-10 |
| *pitx2* | 3.5 | 5.7 | 4.19E-32 | 4.73E-10 |
| *pgd* | 3.4 | 3.7 | 5.77E-131 | 1.25E-37 |
| *lmod3* | 3.4 | 6.6 | 2.68E-02 | 7.63E-04 |
| *st6galnac5* | 3.3 | 3.4 | 2.55E-06 | 3.80E-04 |
| *padi1* | 3.3 | 2.8 | 8.16E-09 | 1.17E-13 |
| *plcd4* | 3.2 | 3.3 | 2.04E-31 | 1.32E-10 |
| *scn2b* | 3.2 | 3.2 | 1.32E-03 | 2.82E-06 |
| *tjp3* | 3.1 | 2.9 | 3.16E-28 | 3.07E-05 |
| *fabp3* | 3.1 | 4.2 | 6.22E-41 | 6.08E-06 |
| *mgp* | 3.0 | 2.3 | 1.19E-18 | 3.11E-06 |
| *vipr2* | 3.0 | 3.2 | 1.26E-09 | 9.82E-06 |
| *bcas1* | 2.9 | 4.3 | 6.99E-11 | 1.40E-17 |
| *actg2* | 2.9 | 9.2 | 1.61E-26 | 1.27E-80 |
| *tmod1* | 2.9 | 2.6 | 4.21E-04 | 6.91E-11 |
| *anxa1* | 2.9 | 5.7 | 8.46E-19 | 8.27E-117 |
| *sh3bgr* | 2.8 | 4.6 | 2.18E-03 | 1.59E-09 |
| *eya2* | 2.8 | 3.6 | 2.44E-03 | 1.09E-19 |
| *fgf1* | 2.8 | 3.0 | 2.98E-08 | 1.41E-05 |
| *nqo1* | 2.7 | 4.1 | 2.79E-15 | 1.03E-28 |
| *gdf10* | 2.7 | 3.0 | 6.84E-09 | 4.61E-06 |
| *musk* | 2.7 | 3.6 | 1.54E-03 | 1.53E-09 |
| *kcnq4* | 2.7 | 3.3 | 7.81E-07 | 1.11E-07 |
| *cyp11a1* | 2.6 | 5.5 | 9.03E-03 | 5.45E-14 |
| *fgfbp1* | 2.6 | 3.2 | 6.72E-16 | 3.81E-15 |
| *phlda1* | 2.6 | 2.8 | 4.76E-08 | 5.40E-27 |
| *nr2f1* | 2.5 | 4.8 | 2.30E-23 | 1.02E-23 |
| *srxn1* | 2.5 | 2.3 | 2.25E-08 | 5.41E-14 |
| *tgm2* | 2.5 | 7.3 | 4.01E-36 | 1.03E-88 |
| *dusp1* | 2.5 | 4.1 | 4.05E-09 | 2.53E-15 |
| *hs3st1* | 2.5 | 4.3 | 5.31E-13 | 2.38E-23 |
| *myom1* | 2.5 | 4.1 | 2.10E-04 | 3.75E-12 |
| *rarb* | 2.2 | 4.0 | 8.12E-15 | 3.16E-16 |
| *mxd1* | 2.1 | 4.2 | 3.29E-36 | 2.70E-46 |
| *ero1a* | 2.1 | 3.6 | 1.04E-22 | 8.41E-46 |
| *ablim3* | 2.0 | 2.9 | 5.37E-12 | 7.25E-14 |
| Notes: Genes with log2 fold change (FC) (expression level in esophagus versus skin)>2 and adjusted P-value (Padj)<0.05 in both chicken and human are listed. The genes are ranked by log2FC in chicken. | | | | |

| **Supplementary Table S8. Categorization of human esophageal epithelial differentiation markers (based on Busslinger et al. 2021*, Suppl. Table S4, Late suprabasal cells)** | | | | |
| --- | --- | --- | --- | --- |
| Gene | Gene category | Enrichment in late suprabasal cells (log2FC) | Padj | Expression level (average number of transcripts per cell) |
| *SPRR3* | EDC | 3.23 | 0.00E+00 | 15.94 |
| *CRNN* | EDC | 2.27 | 1.28E-39 | 0.68 |
| *MAL* | other | 2.25 | 3.26E-64 | 1.11 |
| *KRT4* | KRT | 2.23 | 0.00E+00 | 50.68 |
| *SERPINB3* | other | 2.19 | 8.77E-125 | 2.23 |
| *CSTB* | other | 2.19 | 0.00E+00 | 23.62 |
| *SPRR1A* | EDC | 2.15 | 3.40E-74 | 1.37 |
| *S100A9* | EDC | 2.12 | 5.29E-260 | 11.51 |
| *TGM3* | other | 2.11 | 8.56E-38 | 0.72 |
| *SBSN* | other | 2.06 | 3.67E-41 | 0.81 |
| *S100A8* | EDC | 2.03 | 7.26E-289 | 31.06 |
| *SLURP1* | other | 2.02 | 2.91E-33 | 0.68 |
| *KRT6C* | KRT | 1.94 | 2.54E-169 | 3.57 |
| *CSTA* | other | 1.93 | 4.16E-234 | 15.09 |
| *KRT6B* | KRT | 1.93 | 1.02E-157 | 3.35 |
| *SERPINB1* | other | 1.83 | 2.63E-93 | 2.14 |
| *SPINK5* | other | 1.81 | 4.99E-155 | 3.91 |
| *LYNX1* | other | 1.78 | 2.03E-24 | 0.60 |
| *SPRR2A* | EDC | 1.78 | 2.34E-22 | 0.54 |
| *SLPI* | other | 1.59 | 7.95E-33 | 0.94 |
| *MUC21* | other | 1.58 | 9.54E-12 | 0.35 |
| *SPRR1B* | EDC | 1.53 | 2.07E-69 | 2.08 |
| *RHCG* | other | 1.53 | 1.97E-125 | 5.60 |
| *SERPINB4* | other | 1.50 | 7.79E-12 | 0.38 |
| *CD24* | other | 1.48 | 2.71E-114 | 4.62 |
| *TGM1* | other | 1.43 | 5.55E-11 | 0.37 |
| *GJB2* | other | 1.40 | 2.34E-22 | 0.78 |
| *KRT6A* | KRT | 1.35 | 2.17E-128 | 24.85 |
| *IL1RN* | other | 1.28 | 4.95E-27 | 1.08 |
| *A2ML1* | other | 1.25 | 4.29E-13 | 0.55 |
| *C10orf99* | other | 1.21 | 2.44E-40 | 1.76 |
| *ANXA1* | other | 1.17 | 2.41E-79 | 6.91 |
| *IVL* | EDC | 1.15 | 6.81E-07 | 0.33 |
| *MALL* | other | 1.12 | 7.19E-11 | 0.55 |
| *CLIC3* | other | 1.10 | 1.61E-05 | 0.30 |
| *SCEL* | other | 1.09 | 4.51E-09 | 0.49 |
| *SPRR2D* | EDC | 1.03 | 2.44E-04 | 0.26 |
| *CLCA4* | other | 1.01 | 2.76E-05 | 0.33 |
| *SERPINB13* | other | 1.00 | 7.53E-14 | 0.85 |
| Notes: FC, fold change (late suprabasal versus other cells of human esophagus, single-cell RNA-seq); | | | | |
| Padj, adjusted P value; EDC, epidermal differentiation complex; KRT, keratin. | | | | |
| The table contains all genes with log2FC>1 from Suppl. Table S4, late suprabasal cells. | | | | |
| *Gene expression data are derived and modified from Busslinger GA, de Barbanson B, Oka R, Weusten BLA, de Maat M, van Hillegersberg R, Brosens LAA, van Boxtel R, van Oudenaarden A, Clevers H. Molecular characterization of Barrett's esophagus at single-cell resolution. *Proc Natl Acad Sci U S A.* 2021;118(47):e2113061118. doi: 10.1073/pnas.2113061118 (published under a Creative Commons Attribution 4.0 International License, http://creativecommons.org/licenses/by/4.0/). | | | | |


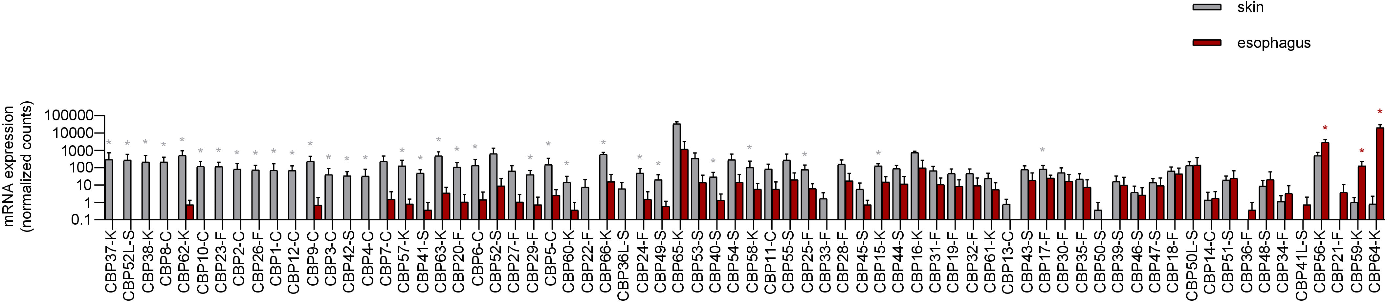


**Supplementary Figure S1. Expression of EDC genes encoding corneous beta proteins (CBPs) in the skin and esophagus of chickens.** The genes were numbered according to their position in the CBP gene subcluster of the EDC as described previously (Lachner et al. 2021). The classification of CBPs in the categories keratinocyte (K), scale (S), claw (C) and feather (F) is indicated by the last letter of the gene name. The genes are ranked according to the ratio of expression levels in the skin versus esophagus. Asterisks mark significant differences (adjusted P-value <0.05) between expression levels in the two organs.
